# Resolution of Cervicogenic Dizziness, Syncope, and Neck Pain Following Restoration of Cervical Lordosis Using Chiropractic BioPhysics® Orthopedic Rehabilitation: A Case Report With One-Year Follow-Up

**DOI:** 10.7759/cureus.114624

**Published:** 2026-08-16

**Authors:** Vicmarie Rivera Ortiz, Jason W Haas, Paul A Oakley, Tim C Norton, Deed E Harrison

**Affiliations:** 1 Chiropractic, Private Practice, Seattle, USA; 2 Research, Chiropractic BioPhysics (CBP) NonProfit, Windsor, USA; 3 Kinesiology and Health Science, York University, Toronto, CAN; 4 Chiropractic, Private Practice, Shoreline, USA; 5 Research, Chiropractic BioPhysics (CBP) NonProfit, Eagle, USA

**Keywords:** cervical lordosis, cervicogenic dizziness, chronic low back pain, chronic neck pain, lumbar lordosis

## Abstract

We report improvements in debilitating cervicogenic dizziness (CD), as well as neck pain and upper and lower back pain, using Chiropractic BioPhysics^®^ (CBP^®^) orthopedic rehabilitation with a one-year follow-up. CD is extremely debilitating and strongly impacts health outcomes. Reports of successful conservative treatments for CD combined with neck and spine pain with improved outcomes and sustained long-term results are rare, and this study adds to the literature. A 29-year-old female suffered from sudden-onset dizziness and frequent syncope elicited by cranio-cervical extension motions. The patient concomitantly reported moderate neck pain, upper mid-back pain, and low back pain, self-reported significant disability, and objectively displayed poor posture and altered spine alignment on radiography. Due to the worsening of her condition, she sought treatment at a facility in Shoreline, WA, USA. Following examination and diagnostic evaluation, she undertook an in-office treatment regimen. The multi-modal orthopedic regimen included postural exercises, postural spinal manipulation, and specific spinal structural traction. This combination Mirror Image (MI®) protocol has been shown to reduce pain and disability in musculoskeletal conditions, with improvement in CD and cervical lordosis. Following 68 in-office treatments over 5.5 months, all initial outcome assessments and physical, orthopedic, and neurological examinations were repeated and recorded. At the post-treatment assessment, the patient reported improvements in CD symptoms and neck, upper back, and lower back pain. Cervical lordosis improved from C2 to C7, measuring +9.4° (kyphotic) to -16.4° (lordotic). At the one-year follow-up, all outcome measures were stable; syncope was fully resolved, and 90% of the previously reported CD symptoms were resolved. Cervical lordosis remained stable at -17.6° (lordotic). This case documents successful treatment in a single patient using conservative spine and posture treatment protocols. This case report followed reporting guidelines. This study may contribute to the evidence aiming to fill the gap in the understanding of CD and potential conservative therapeutic interventions that are directed toward improving spine alignment.

## Introduction

Neck pain is frequently rated as one of the most disabling conditions globally [1]. Patient suffering and economic impact are tremendous, and biomedical research has an interest in investigating effective conservative treatments [2]. Neck pain can be associated with many concomitant conditions, including cervicogenic dizziness (CD). CD is a relatively rare and debilitating condition that is associated with cervical spine positions that disrupt normal sensory and vestibular input and cause dizziness and vertigo, nausea, alterations in heart rate and blood pressure, and can lead to syncope, falls, and serious injury [3-5]. Neck pain and CD are frequently concomitant; however, this case is unique in severity, presentation, and treatment.

CD therapeutic interventions and successful treatment reports are rare; however, recent research is promising [6]. Considering the increased risk of falls and injuries in these patients, they have a greater prevalence of pain syndromes. These pain conditions are highly associated with increased risk of disability and poor health-related quality of life (HRQoL) measures [7]. The aim of this case report is to improve the potential for successful conservative treatment options for CD.

Cervical spine disorders are a significant contributor to pain and poor subjective and objective outcome measures and account for a significant number of years lost due to disability (YLDs) [8]. Neck pain and associated conditions are consistently ranked in the top five causes of disability globally, and more research is needed to elucidate the treatment options for neck pain [9]. Research regarding studies of neck pain coupled with CD successfully treated with conservative methods is rare in the literature [10]. This patient was considered unique by the authors because only cervical extension positions would cause dizziness and frequently led to syncope.

The patient’s unique presentation of CD and syncope was treated using a conservative orthopedic approach that consisted of specific prescribed exercises, specific postural traction derived from the patient’s radiographs, and postural spinal manipulative therapy (SMT). This approach is designed to improve strength of postural and paraspinal musculature, reduce abnormal coronal and sagittal balance, and improve central and peripheral spine neuromusculoskeletal efficiency [11]. This protocol has been published previously in the literature with successful outcomes treating CD and other conditions [12,13]. The treating doctor (V.R.O.) reproduced the multi-modal protocol and had a similar successful outcome. Due to the severity of the condition, the treatment approach, and successful outcome, the patient was selected for potential publication. We present a case report of a single adult female suffering from CD and neck pain that was worsened with cervical extension, who had a successful outcome following a specific rehabilitation approach.

## Case presentation

Ethical statement and Declaration of Helsinki compliance

The patient provided informed consent for evaluation and treatment of their condition prior to examination. The retrospective nature of the study made Institutional Review Board (IRB) approval not applicable in accordance with the U.S. Department of Health and Human Services Common Rule exemption under 45 CFR 46 [14]. Additionally, the Common Rule concerning benign case report investigations of agreeable patients under informed consent makes IRB review unnecessary. This study’s physician and lead author (V.R.O.) obtained patient consent to study the clinically relevant aspect of the condition prior to treatment. The case report does not contain identifiable personal patient data, and the Declaration of Helsinki requirement for further scrutiny is not necessary for this investigation as no patient identifiers are presented. This report was conducted with a strong commitment to ethical integrity, and the data were used in a way that poses no risk to the individual patient.

Patient presentation

A 29-year-old female presented to a chiropractic office with intermittent neck and upper back pain coupled with muscular tension in the neck for two weeks that she rated 8/10 via a paper, handwritten questionnaire. She did not have a specific or traumatic cause of the worsening of neck symptoms. The neck pain had been worsening for two weeks; however, she stated her neck had been an issue for several years, with stiffness reported as the most significant symptom. Further, she reported that she had trained herself not to extend her neck backwards due to symptoms of dizziness, loss of balance, and feelings of passing out. These extension-induced syndromes had been taking place for 14 years, with very occasional bouts of extension-induced syncope due to her limiting the position. She also reported chronic low back pain with a Numeric Rating Scale (NRS) score of 3/10, which worsened with movement, especially bending forward at the torso and neck extension radiating to the lumbar spine [15]. The patient reported taking birth control medication since the age of 15, as well as antidepressants and blood pressure medication that had successfully managed both her depression and hypertension. She reported taking ibuprofen for the last few days as the symptoms worsened, with little to no relief reported.

Prior to any examination, the patient received a consultation with her medical doctor, who had been managing her cardiovascular condition and prescribing blood pressure medication for the prior 14 years, to determine whether any change in cardiovascular status could account for CD and syncope. The medical doctor performed several tests and determined the cause was not vascular in nature and had no explanation for CD and syncope. After the condition did not improve, she sought care from the treating doctor in this case (V.R.O.). The patient's evaluation also found no cause for concern regarding her cardiovascular history or the symptoms that occurred with neck extension. The patient has been previously evaluated by her medical doctor, who prescribed hypertension medications and performed a cardiovascular evaluation to rule out a vascular cause for the extension-related CD. The medical doctor did not discourage her from seeking therapeutic evaluation and treatment for her CD symptoms.

Pre-treatment cervical active range of motion (ROM) exam revealed decreased motion for neck extension (-RxH) at 40°. The cervical extension caused dizziness, sweating, and syncope (normal is 70°) and decreased for right rotation at 70° (normal is 90°) with bilateral 3/10 radiating neck and upper back pain starting in the mid cervical spine and radiating to the upper back. Bilateral cervical lateral flexion ROM was within normal limits (WNL), with 4/10 pain during right lateral flexion in the right trapezius and mild dizziness and 2/10 pain during left lateral flexion in the left trapezius. Thoracolumbar and neck flexion ROM were normal. The following orthopedic tests were positive for pain and dizziness: cervical compression test was 2/10, cervical distraction was 2/10, and George’s cerebrovascular patency maneuver bilaterally caused mild dizziness and visual disturbances. The patient needed to lie down during the exam due to strong dizziness and feelings of fainting while performing cervical extension. Left shoulder depression test measured 4/10. Initial exam included objective outcome measures. The initial Neck Disability Index (NDI) measured 40%, indicating moderate-to-severe disability due to neck pain [15]. The revised Oswestry Disability Index measured 36%, indicating moderate disability due to chronic low back pain [16]. Unfortunately, the Dizziness Handicap Inventory (DHI) score was not obtained prior to treatment.

**Figure 1:**
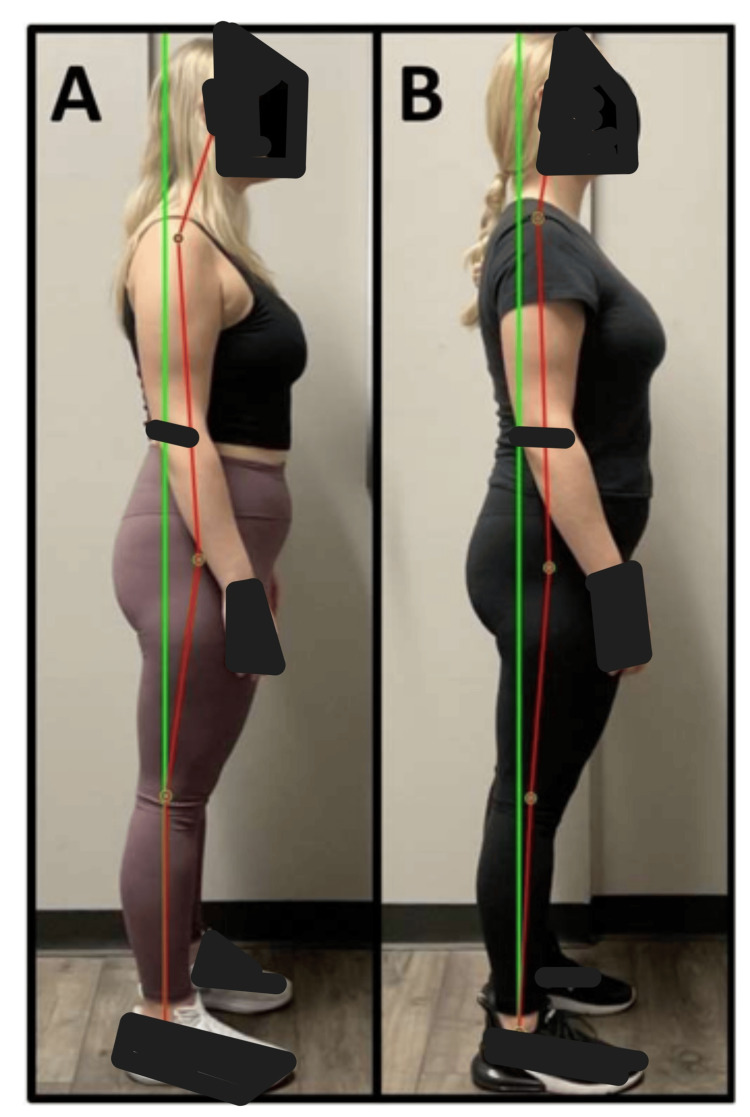
Pre-treatment and post-treatment sagittal posture. Figure A is the initial examination, and Figure B is the post-treatment sagittal posture view. The green line represents a normal, ideal sagittal posture. The red lines represent the actual sagittal posture of the patient. The yellow dotted lines show the thoracic kyphosis and pelvic measurements. The black boxes protect identity and unique physical characteristics.

**Figure 2:**
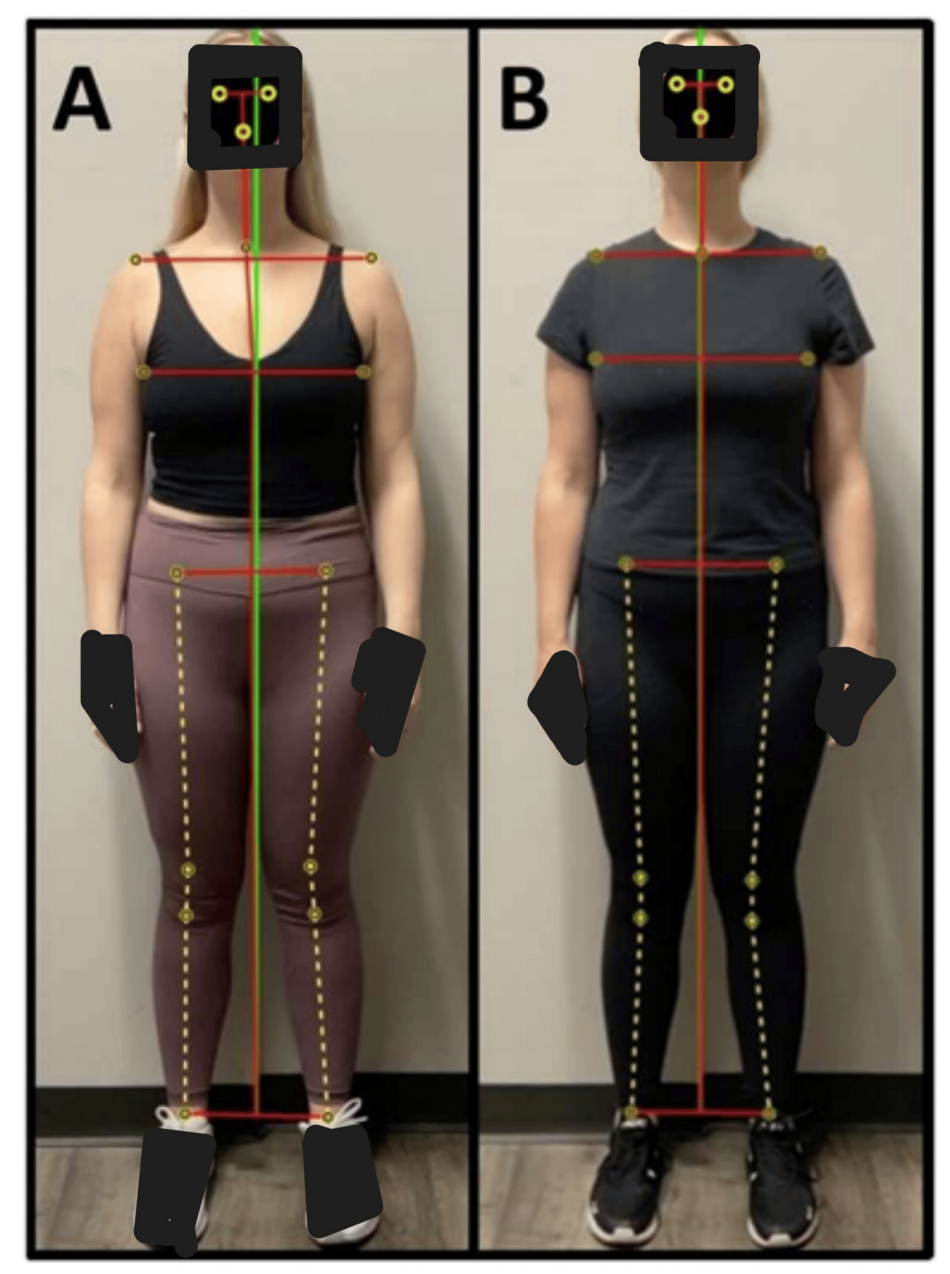
Pre-treatment (left, Figure A) and post-treatment (right, Figure B) coronal posture. The green line represents a normal, ideal coronal posture. The red lines represent the actual coronal posture of the patient. The yellow circles with the black ring inside are anatomical landmarks used to analyze the patient's posture, and the yellow dotted lines show the leg postural alignment. The black boxes protect patient identification.

**Figure 3:**
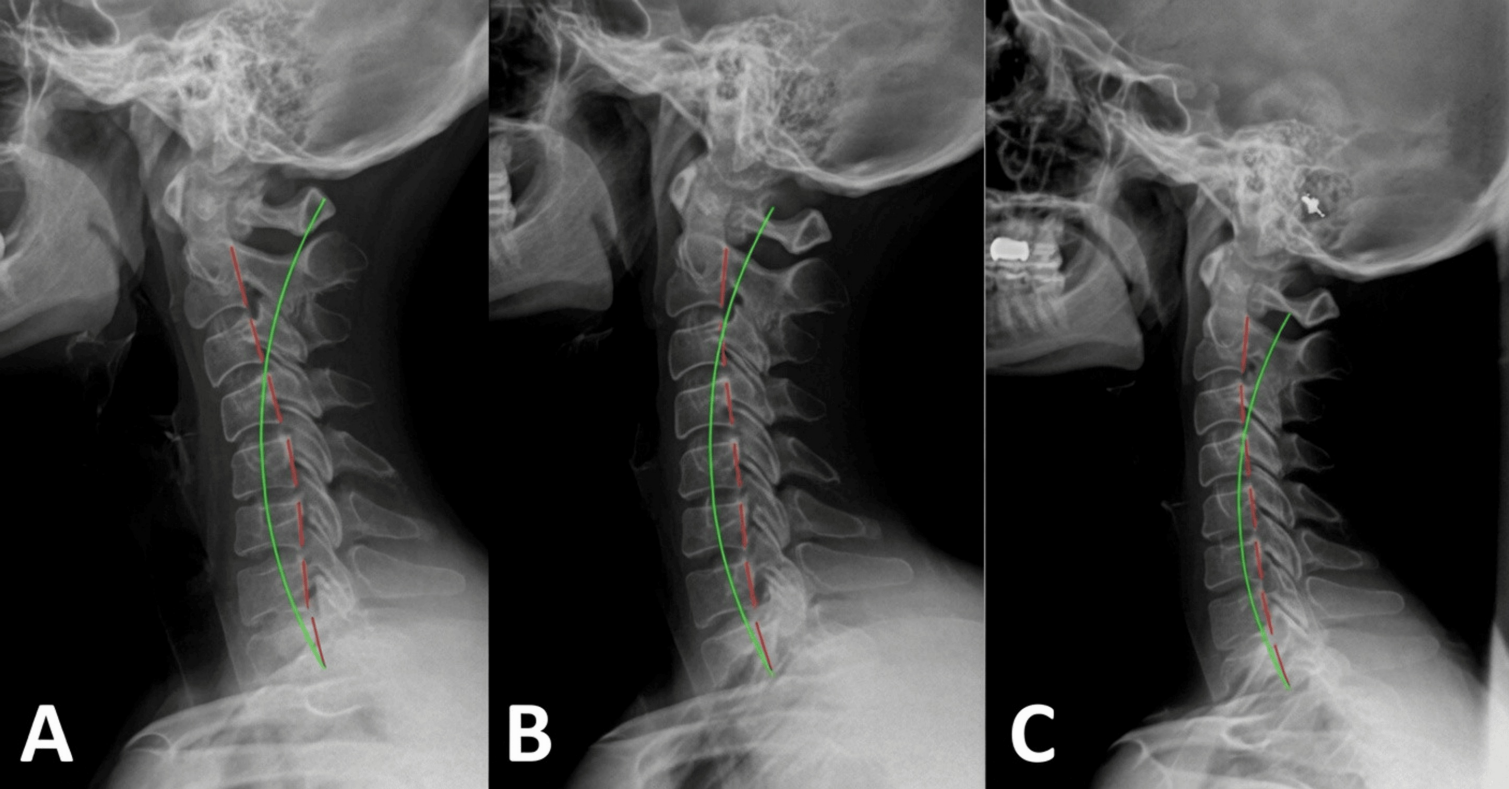
(A) Neutral upright lateral cervical at initial examination. (B) The 5.5-month post-treatment assessment. (C) One-year long-term follow-up. Image features: The green line represents a normal, ideal sagittal lordotic cervical spinal alignment based upon mathematical modeling. The dashed red line represents the vertebral body posterior tangent for segments C2-T1 using the Harrison posterior tangent method. The initial radiograph reveals cervical curve reversal, and post-treatment and long-term follow-up reveal improved lordosis maintained over time. The initial absolute rotation angle measured 9.4° of kyphotic alignment; post-treatment, it measured -16.4° of lordotic alignment; and at the one-year follow-up, it measured -17.6° at C2-C7, indicating that the correction was maintained over time in conjunction with resolution of symptoms.

Initial examination included digital photographic posture analysis using PostureScreen® posture analysis software (PostureCo, Inc., Trinity, FL, USA) [17]. The patient had abnormal posture, including right head translation (+TxH) and anterior head translation (+TzH) (Figure 1, panel A and Figure 2, panel A). Radiographs were obtained and interpreted by a licensed physician in accordance with all state and federal guidelines. No postural alteration beyond the patient’s perceived neutral was performed. All images were acquired using identical radiographic technique and factors. Analysis was performed using PostureRay® X-Ray digitization software (PostureCo, Inc.) [18] using the Harrison posterior tangent method (HPTM). Notable abnormal findings in the cervical spine included an overall kyphotic alignment with a mid-cervical biomechanical buckle at C5-C6. Pre-treatment lateral cervical radiograph revealed an absolute rotation angle (ARA) from C2 to C7 measuring 9.4° (theoretical normal = -29° to -42° [19]), mid-cervical intervertebral posterior tangent angle (relative rotational angle, RRA) from C5 to C6 measuring 6.0° (ideal is -8° per two-segment posterior body tangent [20]), and sagittal translation of C2 with respect to C7 (TzC2-C7) measuring 23.8 mm (ideal is <10 mm [21]) (Figure 3, panel A).

Treatment intervention and outcomes

The patient received 68 in-office treatment sessions over five months, averaging four treatments per week. Patient outcomes were re-evaluated every 36 treatments. Every 12 treatments, the patient completed a written assessment of symptoms, progress, and any concerns. The patient was very compliant, and every assessment found progression and improvement in symptoms. The treatment visits included standard three-region spinal manipulative therapy (SMT). Standard SMT included thoraco-lumbar rotation to end ROM, followed by a gentle pressure in the lumbar spine, poster-anterior pressure along the thoracolumbar spine while prone, and cervical lateral-flexion and contralateral rotation. The SMT may or may not have caused cavitation in the joints, and the manipulations were intended to increase ROM and reduce pain. The patient tolerated the SMT and no adverse events were observed. As the patient’s ROM and pain improved, SMT was utilized less, and the focus moved to postural adjustments.

Chiropractic BioPhysics® (CBP®) Mirror Image® (MI®) corrective postural adjustments, MI® postural exercises, and MI® mechanical traction were utilized for the therapeutic intervention in this case. MI® therapies involve positioning the patient in a corrected or over-corrected postural position to improve sagittal and coronal imbalances and spine alignment. MI® adjustments involved positioning the patient in the opposite position of their abnormal posture on a specialized table and applying adjustments manually by hand using a drop table mechanism, or with an Impulse® Adjusting Instrument (Neuromechanical Innovations®, Chandler, AZ, USA). During the first treatment, the patient experienced moderate dizziness and syncope symptoms during cervical extension, and the patient fainted twice that day, without injury and on a flat table. This further confirmed that the patient was experiencing a vasovagal syncope reflex activated by cervical extension. Extension MI® protocols are used to restore cervical lordosis, and precautions were made to accommodate the procedures without syncope. Three steps were identified to prevent losing consciousness and were applied as a safety method during cervical extension in MI® therapies: (1) the patient’s legs were elevated 30 cm above the heart level; (2) the patient was instructed to make a fist or squeeze a rubber ball to increase peripheral vascular resistance; (3) the patient remained supine until symptoms resolved and then stood up slowly.

**Figure 4:**
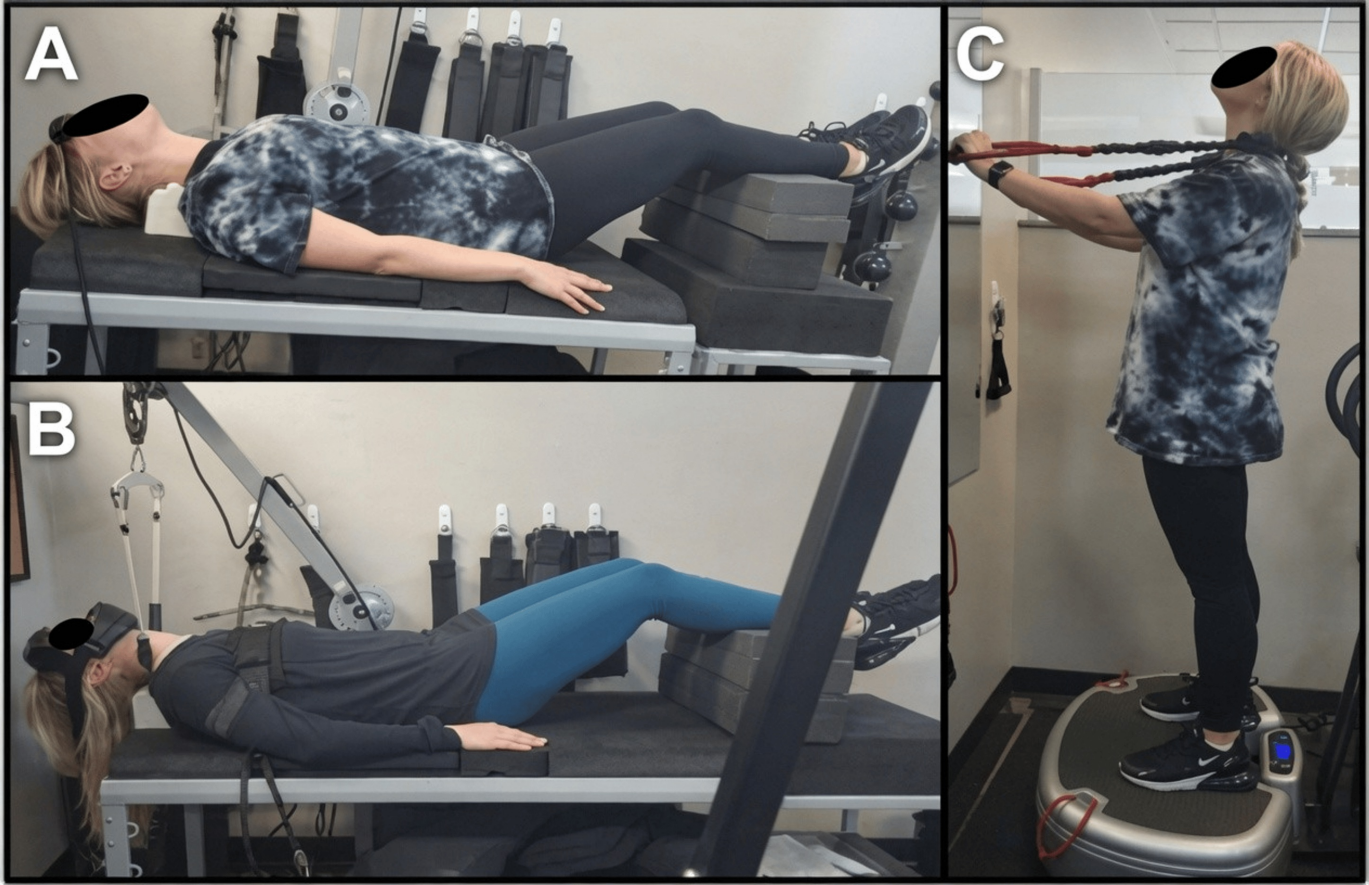
(A) Mirror Image® traction using 3-D DennerollTM Traction Table System and Cervical DennerollTM. (B) Mirror Image® traction using 3-D DennerollTM Traction Table System. (C) Mirror Image® exercise using CBP® Pro-Lordotic Neck Exerciser and Power Plate® whole-body vibration platform (Northbrook, IL, USA).

MI® mechanical traction was performed using the 3-D Denneroll^TM^ Traction Table System (Denneroll^TM^ Spinal Orthotics, New South Wales, Australia) with a medium Cervical Denneroll^TM^ Spinal Orthotic (Denneroll^TM^ Spinal Orthotics) placed at the mid-to-lower cervical spine and a forehead strap holding the head on the table, preventing head extension (Figure 4A). At visit 24, the MI® traction increased in intensity by adding a posterior-to-anterior force in the mid-cervical spine to increase bending moment into lordosis (Figure 4B). Additionally, the patient protocol included extension exercises (described below) (Figure 4C).

At the 24th visit, a progress evaluation revealed the patient was able to perform cervical extension with no dizziness or syncope; therefore, the Fedorchuk lordosis-inducing procedure (FLIP) exercise was added to the treatment regimen [22,23]. This exercise is designed to increase strength and endurance in the cervical paraspinal musculature while inducing cervical lordosis by performing a series of movements in a particular order: (1) maximum anterior head translation +T_z_^H^, which causes an intersegmental coupling pattern of the cervical spine, creating lordosis of the upper cervical spine and kyphosis of the lower cervical spine; (2) while maintaining +T_z_^H^, maximum -R_x_^H^, which allows for the upper cervical spine to maintain its lordosis and for the lower cervical spine to move toward a healthy lordosis; (3) while maintaining the -R_x_^H^, posterior and inferior head translation (-T_z_^H^ and -T_y_^H^), allowing for the head to return to a normal postural position while maintaining the induced cervical lordosis from previous movements.

The patient was instructed to perform this exercise initially for three to five repetitions at minimal intensity, and the final position was held for 5-10 seconds. She was instructed to increase the intensity of the exercise to patient tolerance with no more than 25 exercises performed in-office and at home daily. The patient was instructed to work the muscles to only mild soreness, and if the soreness became too intense, to reduce the frequency and duration of the FLIP exercises.

MI® traction was progressed by extending the patient’s head over the medium cervical Denneroll^TM^ and off of the table and then further with 4.5 kg hanging from her head with a mid-cervical strap to improve cervical curve (Figure 4B). The patient was also prescribed left head translation (+T_X_^H^) combined with left torso translation (-T_X_^T^) and cervical extension (-R_X_^H^) MI® exercises for coronal posture correction on a whole-body vibration (WBV) platform to increase blood circulation (Figure 4C). All MI® traction protocols initially began with one to three minutes in the position, and the patient was observed for at least 24 hours before repeating the traction. The time increased gradually from three minutes to patient tolerance. The patient was able to tolerate the traction for 10 minutes after the 35th visit comfortably and with no adverse reactions. She was also able to tolerate the traction up to four times per week and was able to tolerate 20 minutes of traction by the final in-office treatment.

At 36 treatments, reassessment of the initial examination was performed. Post-treatment posture analysis showed improvement in posture (Figures 1B, 2B). Post-treatment radiographic examination revealed improvement in ARA C2-C7 to -16.4°, RRA C5/6 to 1.5°, and Tz C2-C7 to 8.9 mm (Figure 3B). Post-treatment patient-reported outcomes (PROs) revealed improvement in NDI to 0%, indicating no disability due to neck pain, and Oswestry Disability Index (ODI) to 2%, indicating minimal-to-no disability due to low back pain. The patient reported she no longer experienced dizziness or syncope during cervical extension. No other symptoms or conditions were reported at the post-treatment follow-up. She reported very high satisfaction with her treatment and outcomes.

A one-year follow-up examination was conducted, and the initial examinations were repeated. The patient did not receive any in-office traction during the time between post-treatment and long-term evaluations. She reported doing her exercises and home Denneroll traction two times per month. The patient reported no new injuries, no CD symptoms, no syncope, and no other abnormal health conditions. The PROs were found to be stable in the long term and very mild. The NDI and ODI were both reported to be 4%, indicating very minimal disability due to pain. Cervical lordotic curvature was well maintained, and ARA C2-C7 measured -17.6°, and the sagittal balance was well preserved, measuring 8.8 mm from C2 to C7. The lordotic cervical curvature appeared stable at long-term follow-up (Figure 3C). Table 1 presents the pre- and post-treatment outcomes of note. At long-term follow-up, all post-treatment outcomes were well maintained. The NRS score for neck pain satisfied the requirement for a minimally important clinical difference from pre-treatment to post-treatment and was maintained at long term [24].

**Table 1:** The patient's results from the initial, post-treatment, and long-term follow-up examinations and orthopedic testing, pain scales, range of motion, outcome questionnaires, and cervical radiography are presented. Sx. = symptoms; NRS = Numeric Rating Scale; Cerv. = cervical; Rt = right; Lt = left; Lat = lateral; Flex = flexion; Ext = extension; SLR = straight leg raising test; NDI = Neck Disability Index; RODI = revised Oswestry Disability Index; ARA = absolute rotation angle. Additional radiographic and other results are available upon request.

Exam	Initial	Post-treatment	Long-term follow-up
NRS (Pain)	Cervical = 8/10	Cervical = 0/10	Cervical= 0/10
	Thoracic = 3/10	Thoracic= 1/10	Thoracic = 1/10
	Lumbar = 3/10	Lumbar = 1/10	Lumbar = 0/10
Ranges of motion	Cerv ext. = 40 deg., fainting, dizzy	Cerv ext. = 60 deg. 0/10, no Sx.	Cerv. Ext. = 60deg. 0/10, no Sx.
	Cerv. Rt rot. = 70 deg., 3/10 pain	Cerv. Rt rot. = 80 deg., 0/10 pain	Cerv. Rt rot. = 80 deg., 0/10 pain
	Cerv Rt lat. flex. = 4/10 pain & dizzy	Cerv Rt lat. flex. = 0/10 pain	Cerv. Rt lat. flex. = 0/10 pain
	Cerv Lt lat. flex. = 2/10 pain	Cerv Lt lat. flex. = 0/10 pain	Cerv. Lt lat. flex. = 0/10 pain
Orthopedic test	Cerv. compression = 4/10	Cerv. compression = 0/10	Cerv. compression = 0/10
	Cerv. distraction = 2/10 pain	Cerv. distraction = 0/10	Cerv. distraction = 0/10
	Lt shoulder depression = 4/10	Lt shoulder depression = 0/10	Lt shoulder depression = 0/10
	George’s maneuver = dizziness	George’s maneuver = negative	George's maneuver = negative
	SLR = 2/10, low back pain	SLR = negative	SLR = negative
Questionnaires	NDI = 40%	NDI = 0%	NDI = 4%
	RODI = 36%	RODI = 2%	RODI = 4%
Radiography			
C2-C7 ARA	9.4° (kyphotic)	-16.4° (lordotic)	-17.6° (lordotic)
C2-C7 Translation	23.8 mm	8.9 mm	8.8 mm

## Discussion

This case documents the successful reduction of dizziness in a 29-year-old patient who also suffered from chronic neck pain and upper and lower back pain and experienced episodes of syncope. The first re-evaluation was completed at 36 treatments and reassessed following 68 treatments over a five-month period; an approximate 26° increase in cervical lordosis was achieved. A one-year follow-up assessment confirmed that the curve restoration remained stable with home traction twice a month, as was the patient’s resolution of dizziness and syncope symptoms.

This case is consistent with the results of a recent randomized trial by Moustafa et al. [6], who determined that patients having cervical hypolordosis and dizziness could be successfully treated by a multimodal program including cervical extension to increase cervical lordosis. This had to be approached very carefully, as the patient had trained herself not to extend, as it was the single cause of her CD. This movement of the neck causing CD is consistent with other studies of CD. In the Moustafa et al. study, it was found that following 30 treatments, a sample of 36 patients achieved a 14° increase in cervical lordosis (as well as a 25 mm reduction in anterior head carriage) that resulted in an improved dizziness frequency, severity, and a 24-point reduction on the DHI [6]. This case is also consistent with the cases reported by Gerstin et al. [13] and Fortner et al. [25], who also demonstrated improved dizziness symptoms in patients who received CBP extension traction to the cervical spine. As in this case, there may be a cautious progression toward achieving full cervical extension in patients with this condition [13].

This case achieved a greater increase in lordosis (≈26°) than that reported in the Moustafa et al. randomized trial (14°) [6] and in the Gerstin et al. (19.5°) [13] and Fortner et al. (13°) [25] case reports. This report is the only known case to demonstrate the correction in a patient presenting with dizziness, syncope, and initial cervical kyphosis. It is important to consider that patients presenting with cervical kyphosis may require more treatment sessions of extension traction because a greater degree of extension is needed to achieve correction of cervical lordosis. As previously reported by Norton et al. [26], they would also need to be treated on a maintenance basis thereafter indefinitely to prevent a regression of the initial curve correction. Ideally, this patient and similar other candidates would continue to perform maintenance traction procedures at a frequency of twice monthly after the initial intensive treatment regimen at minimum. Long-term management after successful outcomes should entail random radiographic assessment to assess the stability of the initial correction [25]. Home care is highly encouraged for all abnormal spine conditions involving abnormal spine alignment and patient participation, and compliance has previously been reported as beneficial to long-term outcomes. Severe spine conditions should require provider integration and communication in the event of any worsening of CD symptoms, or syncope leading to unconsciousness, falls, or other trauma.

It has previously been postulated that dizziness may result from alteration or perturbation of information from afferent sensory structures of the cervical spine [27,28]. These afferent sensory structures play a primary role in the timing and coordination of key reflexes involved in balance and smooth muscle function. These reflexes, including the tonic neck reflex, cervicocollic reflex, and ocular motor reflex, are strongly associated with the proper function of the vestibular system, and abnormalities in these systems can confirm the diagnosis of CD [28,29]. In the trial by Moustafa et al. [6], since the control group had a regression of symptoms following the cessation of treatment, while the treatment group, who had an increase in cervical curvature from extension traction, maintained their dizziness symptom relief, it was suggested that the loss of cervical lordosis was a causative factor in the etiology of dizziness symptoms.

How would loss of cervical lordosis be a key factor in eliciting dizziness? As argued by Fortner et al. [25], the loss of cervical lordosis is associated with lengthening of the spinal canal; this, in turn, may cause a traction effect on the spinal cord [30-32]. Normal head movements may then cause a dynamic strain on the neural structures; thus, normally natural physiological head motions may now elicit pathologic strain on the pons-cord tract. Indeed, head motion is intrinsically linked to neurologic structures [33,34], and one mechanistic link may be via dynamic overstrain. Microstructural damage to the delicate neural structures may indeed result in dizziness symptoms in certain circumstances [35]. The possible link to alleviating the dizziness associated with loss of lordosis is to implement procedures directed at increasing the cervical curve. Although there are cases reported in the manual therapy literature of treatments used for treating CD [36], there is a lack of evidence on the long-term effectiveness, and none includes syncope. The physiotherapeutic goal of increasing cervical lordosis is a novel treatment approach for patients suffering from dizziness and having cervical hypolordosis/kyphosis.

Limitations of this case include representing only a single patient. However, the complication of syncope associated with cervical extension, which is necessary to improve cervical lordosis, makes this case unique as it represents a challenging clinical scenario [37]. Further, no scale was used to document the magnitude of dizziness symptoms; the implementation of the DHI [38] would have added to the strength of the case and should be recommended for future studies. At the time of the study, the treating doctor was unaware of the DHI. More research is necessary to assess the validity of cervical curve correction as a treatment for patients suffering from the combination of dizziness and neck pain. Additionally, as this is a single case report, other reasons for improvement include natural symptom/disease fluctuations and non-specific treatment effects. Finally, as a single case report, these findings cannot establish efficacy or causality, and larger controlled studies are required.

## Conclusions

This case presents the successful resolution of positional CD and syncope in a young female patient who presented with a cervical kyphosis. Symptom relief was achieved and maintained at one-year follow-up through a multimodal CBP® rehabilitation program that restored cervical lordosis. The results align with the limited existing literature on cervical curve correction for CD and suggest that improving sagittal alignment may be an important therapeutic target. Further research incorporating spinal biomechanical analysis is warranted to confirm these findings in patients with vasovagal syncope and CD.

## References

[1] Kazeminasab S, Nejadghaderi SA, Amiri P (2022). Neck pain: global epidemiology, trends and risk factors. BMC Musculoskelet Disord.

[2] Castellini G, Pillastrini P, Vanti C (2022). Some conservative interventions are more effective than others for people with chronic non-specific neck pain: a systematic review and network meta-analysis. J Physiother.

[3] De Hertogh W, Micarelli A, Reid S, Malmström EM, Vereeck L, Alessandrini M (2025). Dizziness and neck pain: a perspective on cervicogenic dizziness exploring pathophysiology, diagnostic challenges, and therapeutic implications. Front Neurol.

[4] Magnusson M, Malmström EM (2016). The conundrum of cervicogenic dizziness. Handb Clin Neurol.

[5] De Vestel C, Vereeck L, Van Rompaey V, Reid SA, De Hertogh W (2022). Clinical characteristics and diagnostic aspects of cervicogenic dizziness in patients with chronic dizziness: a cross-sectional study. Musculoskelet Sci Pract.

[6] Moustafa IM, Diab AA, Harrison DE (2017). The effect of normalizing the sagittal cervical configuration on dizziness, neck pain, and cervicocephalic kinesthetic sensibility: a 1-year randomized controlled study. Eur J Phys Rehabil Med.

[7] Gashi AI, Azemi A, Kovačič T (2025). A cross-sectional analysis of pain, neck disability, functional performance, and quality of life in patients with cervical spondylosis. J Clin Med.

[8] Ferrer-Peña R, Vicente-de-Frutos G, Flandez-Santos D, Martín-Gómez C, Roncero-Jorge C, Calvo-Lobo C (2019). Patient-reported outcomes measured with and without dizziness associated with non-specific chronic neck pain: implications for primary care. PeerJ.

[9] Wu H, Li Y, Zou C, Guo W, Han F, Huang G, Sun L (2025). Global burden of neck pain and its gender and regional inequalities from 1990 - 2021: a comprehensive analysis from the Global Burden of Disease Study 2021. BMC Musculoskelet Disord.

[10] Vural M, Karan A, Albayrak Gezer İ (2021). Prevalence, etiology, and biopsychosocial risk factors of cervicogenic dizziness in patients with neck pain: a multi-center, cross-sectional study. Turk J Phys Med Rehabil.

[11] Anwar G, Moustafa IM, Ahbouch A, Alrahoomi A, Harrison DE (2025). The magnitude of sagittal head posture displacement and patient demographics predict rehabilitation outcomes in patients with chronic nonspecific neck pain. Sci Rep.

[12] Moustafa IM, Diab AA, Harrison DE (2022). The efficacy of cervical lordosis rehabilitation for nerve root function and pain in cervical spondylotic radiculopathy: a randomized trial with 2-year follow-up. J Clin Med.

[13] Gerstin G, Oakley PA, Harrison DE (2020). The treatment of dizziness by improving cervical lordosis: a Chiropractic BioPhysics® case report. J Phys Ther Sci.

[14] Walch-Patterson A (2020). Exemptions and limited institutional review board review: a practical look at the 2018 Common Rule requirements for exempt research. Ochsner J.

[15] Begum R, Parikh P, Walton D, Rushton A (2026). Physical measures of physical functioning as prognostic factors in predicting outcomes for neck pain: protocol for a prospective longitudinal cohort study. PLoS One.

[16] Powers JH 3rd, Ballinger R, De Palma A, de la Cruz M, Howard K (2026). Assessment of the content validity of the Oswestry Disability Index (ODI) in patients with degenerative disc disease (DDD): a qualitative study. BMC Musculoskelet Disord.

[17] Boland DM, Neufeld EV, Ruddell J, Dolezal BA, Cooper CB (2016). Inter- and intra-rater agreement of static posture analysis using a mobile application. J Phys Ther Sci.

[18] Fedorchuk C, Comer RD, McRae C, Bak D, Lightstone DF (2023). Validity of radiographic analyses between hand-drawn and computer- aided measurements: a double-blinded test-retest trial. Curr Med Imaging.

[19] Oakley P, Sanchez L, Harrison D (2021). Medical radiologists may not consider the cervical lordosis in radiology reports: a comparison of subjective qualitative assessment versus objective quantitative mensuration in 100 consecutive patients at one medical imaging center. J Contemporary Chiro.

[20] Harrison DD, Janik TJ, Troyanovich SJ, Holland B (1996). Comparisons of lordotic cervical spine curvatures to a theoretical ideal model of the static sagittal cervical spine. Spine (Phila Pa 1976).

[21] Harrison DD, Harrison DE, Janik TJ, Cailliet R, Ferrantelli JR, Haas JW, Holland B (2004). Modeling of the sagittal cervical spine as a method to discriminate hypolordosis: results of elliptical and circular modeling in 72 asymptomatic subjects, 52 acute neck pain subjects, and 70 chronic neck pain subjects. Spine (Phila Pa 1976).

[22] Fedorchuk C (2017). Cervical coupling patterns following head retraction with compression to neutral: a prospective study. Proceedings of the 39th CBP Annual Convention.

[23] Fedorchuk C, Lightstone DF, Comer RD (2019). Radiographic stress analysis to determine the proper coupling patterns of the cervical spine prior to intervention. Proceedings of the 2nd International Conference on Medical Imaging and Case Reports (MICR).

[24] Monticelli A, Van Grootven B (2025). Exploring established cut-off points for pain levels in the numeric rating scale: insights from a literature overview. Pain Manag Nurs.

[25] Fortner MO, Oakley PA, Harrison DE (2018). Alleviation of posttraumatic dizziness by restoration of the cervical lordosis: a CBP® case study with a one year follow-up. J Phys Ther Sci.

[26] Norton TC, Oakley PA, Haas JW, Harrison DE (2026). Long-term stability of reducing cervical kyphosis via Chiropractic BioPhysics® extension traction procedures: a case series. J Phys Ther Sci.

[27] Li Y, Yang L, Dai C, Peng B (2022). Proprioceptive cervicogenic dizziness: a narrative review of pathogenesis, diagnosis, and treatment. J Clin Med.

[28] Kristjansson E, Treleaven J (2009). Sensorimotor function and dizziness in neck pain: implications for assessment and management. J Orthop Sports Phys Ther.

[29] Reiley AS, Vickory FM, Funderburg SE, Cesario RA, Clendaniel RA (2017). How to diagnose cervicogenic dizziness. Arch Physiother.

[30] Breig A (1978). Adverse Mechanical Tension in the Central Nervous System: An Analysis of Cause and Effect: Relief by Functional Neurosurgery. Almqvist & Wiksell International.

[31] Panjabi M, White A 3rd (1988). Biomechanics of nonacute cervical spinal cord trauma. Spine (Phila Pa 1976).

[32] Breig A (1960). Biomechanics of the Central Nervous System. Uppsala: Almqvist & Wiksell International.

[33] Moustafa IM, Youssef A, Ahbouch A, Tamim M, Harrison DE (2020). Is forward head posture relevant to autonomic nervous system function and cervical sensorimotor control? Cross sectional study. Gait Posture.

[34] Welgampola MS, Bradshaw AP, Halmagyi GM (2019). Assessment of the vestibular system: history and physical examination. Adv Otorhinolaryngol.

[35] Fife TD, Giza C (2013). Posttraumatic vertigo and dizziness. Semin Neurol.

[36] Lystad RP, Bell G, Bonnevie-Svendsen M, Carter CV (2011). Manual therapy with and without vestibular rehabilitation for cervicogenic dizziness: a systematic review. Chiropr Man Therap.

[37] Aydin MA, Salukhe TV, Wilke I, Willems S (2010). Management and therapy of vasovagal syncope: a review. World J Cardiol.

[38] Jacobson GP, Newman CW (1990). The development of the Dizziness Handicap Inventory. Arch Otolaryngol Head Neck Surg.

